# Non-Enzymatic Hydrolysis of RNA in Workers of the Ant *Nylanderia pubens*

**DOI:** 10.1673/031.012.14601

**Published:** 2012-12-15

**Authors:** Steven M. Valles, Charles A. Strong, Eileen A. Buss, David H. Oi

**Affiliations:** ^1^ USDA-ARS Center for Medical, Agricultural and Veterinary Entomology, 1600 SW 23^rd^ Drive, Gainesville, FL 32604; ^2^ University of Florida, Department of Entomology and Nematology, Gainesville, FL 32611

**Keywords:** Invasive species, nucleic acid degradation, RNA purification

## Abstract

During preparation of total RNA from *Nylanderia pubens* (Forel) (Hymenoptera: Formicidae) workers for use in expression library construction, severe RNA degradation consistently occurred. This degradation was masked by spectrophotometric analysis but clearly evident by microfluidic-based assay. Although not specifically identified, the degrading entity was endogenous and localized to the abdomen (terminal abdominal segments) of adult ants. RNA degradation was not observed in preparations of larvae, non-melanized pupae, or eggs. Various RNase and protease inhibitors had no protective effect. However, the metal chelating agent ethylenediaminetetraacetic acid prevented RNA degradation and provides insight into the occurrence.

## Introduction

*Nylanderia pubens* (Forel) (Hymenoptera: Formicidae), previously *Paratrechina pubens* (LaPolla, Brady, and Shattuck 2010), is an invasive ant species that in recent years has developed into a serious pest ant problem in the United States and Caribbean (Wetterer and Keularts 2008; MacGown and Layton 2010). The rapidly expanding range and explosive, localized population growth exhibited by this ant have quickly elevated it to pest status, with professional entomologists and the pest control industry scrambling to understand its biology and develop effective control methods (Drees et al. 2009; Warner and Scheffrahn 2010). A dearth of information exists concerning the biology of this ant. Indeed, only a single non-taxonomic-based scientific study on *N. pubens* was identified (Cook et al., 2010). During preparation of total RNA from adult *N. pubens* for use in expression library construction to screen for pathogens, severe RNA degradation was observed. This degradation was masked by spectrophotometric analysis but clearly evident by microfluidic-based assay. Thus, the objective of this paper was to document and characterize this endogenous RNA-degrading entity in order to make those interested in molecular-based studies with this ant species aware of its existence and the consequences of its presence in down-stream assays.

## Materials and Methods

### RNA extractions

RNA was extracted from different developmental stages of *N. pubens* (eggs, larvae, non-melanized pupae, and adults) using the guanidium isothiocyanate method (specifically, Trizol (Invitrogen, http://www.invitrogen.com/) according the manufacturer's instructions). *N. pubens* were obtained from colonies collected from field sites located in Gainesville, Alachua County, Florida, and maintained in the laboratory. Colonies were housed in nesting tubes described by Oi and Williams (2003) and reared on a diet of frozen crickets, live housefly larvae, and a 10% sucrose solution. Ants were taken alive from a colony by featherweight forceps, placed directly into a 1.5 mL microcentrifuge tube containing 500 µl of Trizol solution, and homogenized for 20 seconds by hand with an RNase-free plastic pestle. Ten individuals of each stage (adult, larvae (mixture of instars), non-melanized pupae), or approximately 100–200 eggs, were used in each preparation. Chloroform (200 µl) was added to the homogenate, which was vortexed for 30 seconds and centrifuged at 12,000 × g for 5 minutes. The RNA-containing supernatant (∼150 µl) was precipitated with 2-propanol (1 mL) and centrifuged at 12,000 × *g* for 5 minutes. The pellet was washed with 70% ethanol and resuspended in 15–30 µl of nuclease free water (Ambion, Invitrogen). RNA was also extracted from tagma of adult ants: head and thorax, abdomen, and the last 3 to 4 terminal abdominal segments. Homogenization of whole adults was also conducted in different solutions (150 µl) just before the addition of Trizol. These solutions included ethylenediaminetetraacetic acid (EDTA) (5 mM, 50 mM, 500 mM), neutralizing TRISHCl buffer (100 mM, pH 9), RNase Out (Invitrogen; 2.5, 5, 10 µl), proteinase K (20 and 200 µg), diethyl pyrocarbonate (DEPC, 100 µl; Sigma), and formic acid (0.1 and 1 mM).

### RNA analyses and RT-PCR

RNA quality was assessed by microfluidic analysis on an Agilent 2100 Bioanalyzer (Agilent, http://www.home.agilent.com/), using the RNA 6000 Nano kit according to the manufacturer's directions. Microfluidic assays were completed immediately after RNA extraction using a 1 µl volume of purified sample. RNA size standards (200 to 6,000 nucleotides) and a positive RNA control (larval *N. pubens* RNA of known integrity) were included for each experiment. For comparison, RNA quality and quantity were determined spectrophotometrically on an ND-1000 spectrophotometer (Nanodrop Technologies, Inc., http://www.nanodrop.com/). The 260:280 nm ratio and quantity of RNA were determined.

RNA integrity was evaluated by its ability to serve as a template for transcription into cDNA and subsequent amplification by PCR. Oligonucleotide primers were designed for the *N. pubens* housekeeping gene, ubiquitin. RNA (50 ng) from different preparations and life stages was digested with DNase I (New England Biolabs, http://www.neb.com/) for 10 minutes at 37° C according to the manufacturer's instructions. The DNase-digested RNA was reverse transcribed with Superscript III reverse transcriptase (Invitrogen) at 55° C for 30 minutes using oligonucleotide primer p1222 (5′ TGCAATAGCAATAGTGTCGTTGCTATAAACAGGT). PCR was subsequently conducted with Platinum Taq polymerase (Invitrogen) and oligonucleotide primers p1222 and p1221 (5′ TGCCTCAGTTAATGACACGTCAGAAAATTCGA) using the following program: 94^°^C for 2 minutes, 35 cycles of 94° C for 15 seconds, 62^°^C for 15 seconds, and 68° C for 30 seconds, followed by a polishing step of 68° C for 5 minutes. Amplicons were separated on a 1% Agarose gel and visualized with SYBR-safe (Invitrogen). DNase digestion was verified by including the undigested RNA as template in PCR. Non-template controls and positive controls (typically from larval RNA) were included in each evaluation.

## Results and Discussion

**Figure 1.**
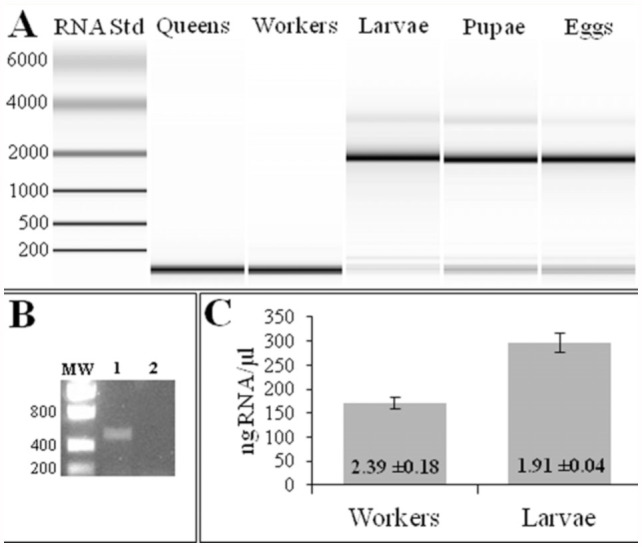
(A) Microfluidic analyses of total RNA prepared from different life stages of *Nylanderia pubens* by the Trizol method. RNA standards and their corresponding sizes are provided in the left-most column. (B) PCR of a portion of the *N. pubens* ubiquin gene. DNase-treated RNA (from panel A) of larvae (lane 1 ) and adults (lane 2) was reverse transcribed and subsequently amplified by PCR. MW = molecular weight markers. Sizes shown on the left. (C) Results of spectrophotometric analysis of the RNA preparations from workers and larvae from the upper panel on an ND-1000 spectrophotometer. Standard deviation values are indicated by error bars. The mean 260:280 nm ratio is provided for each life stage within the bar. High quality figures are available online.

The integrity of RNA purified from different life stages of *N. pubens* with Trizol reagent yielded consistent results; RNA from workers, queens, and alates was completely degraded, while RNA from larvae, pupae, and eggs was not (Figure 1A). The microfluidic RNA assay separates RNA by mass and scores the integrity of the RNA based on comparative peaks at the known 18S and 28S rRNA subunits exhibited by eukaryotes. However, the 28S rRNA subunit of many insects has been shown to contain two hydrogen-bonded fragments (a “hidden break”) that dissociate upon heating or during the purification process and co-migrate with the 18S subunit (Winnebeck, Millar, and Warman 2010). Thus, gel electophoretic profiles of insect total RNA typically have a single peak at the 18S position. Based on microfluidic separation of RNA purified from larvae, pupae, and eggs, *N. pubens* conforms to this hidden break characteristic. Figure 1A illustrates a very weak band at approximately 3,500 nucleotides (larvae, pupae, eggs) which corresponds to intact 28S rRNA. A corresponding strong band was detected at approximately 2,000 nucleotides, the 18S rRNA subunit. Conversely, electrophoretic separation of RNA from adult stages exhibited a strong band at approximately 50–100 nucleotides without any detectable bands in the 18S and 28S positions. Thus, total RNA from worker and queen stages of *N. pubens* appeared completely degraded and of questionable use in any downstream molecular biology assay (e.g., reverse transcription polymerase chain reaction or library construction). Indeed, RT-PCR of the housekeeping gene, ubiquitin of *N. pubens*, failed to generate an amplicon from DNase-treated RNA templates in adult workers, confirming the denatured state of this RNA (Figure 1B).

The initial conclusion in response to this result was that careless laboratory procedures were introducing an RNase into the preparation and causing degradation. However, multiple replications (> 20) of the purification procedure (by different individuals and from different ant colonies) always resulted in degraded RNA from adult stages and intact RNA from immature stages. Interestingly, spectrophotometric analysis masked the degraded nature of the RNA (Figure 1C). The spectrophotometric analysis of worker and larval RNA preparations yielded 260:280 nm ratios near 2 and measurable concentrations (above 150 ng RNA/µl), which are traditional indicators that the preparation is relatively free of contaminating protein and of acceptable quality (Sambrook and Russell 2001). However, the microfluidic analysis of these samples (Figure 1A) indicated that they were in fact degraded severely.

**Figure 2.**
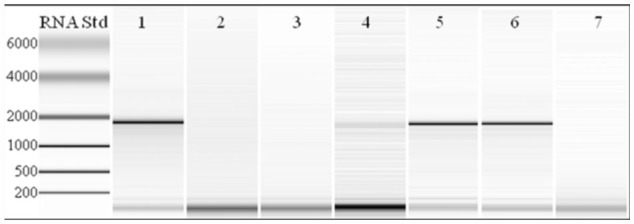
Microfluidic analyses of total RNA prepared from different tagma of *Nylanderia pubens* by the Trizol method. Lane 1, head and thorax (adults); lane 2, abdomen (adults); lane 3, whole body (adults); lane 4, terminal abdominal segments (adults); lane 5, remaining abdominal segments (adults); lane 6, whole body (larvae); lane 7, whole body (larvae) and terminal abdominal segments (adults). High quality figures are available online.

These experiments certainly suggested that an entity was being liberated from adult ants during homogenization, causing RNA degradation. In an effort to localize this substance or, otherwise, identify the conditions causing the degradation, RNA from different tagma was extracted by the Trizol method and evaluated. RNA extracted from the head and thorax of *N. pubens* exhibited a strong 18S fragment band, indicating that the integrity of the sample was intact (Figure 2, lane 1). Conversely, RNA extracted from the abdomen (Figure 2, lane 2) was completely degraded. When the last 3 or 4 terminal abdominal segments of the abdomen were extirpated and used as starting material for RNA purification, the RNA was similarly degraded. However, the remaining portion of the abdomen (i.e., without the terminal abdominal segments) yielded intact RNA (Figure 2, lane 6). Experiments in which a homogenate prepared from terminal abdominal segments was added to intact RNA preparations resulted in the prompt degradation of this RNA (Figure 2, lanes 6 (without abdominal homogenate) and 7 (with abdominal homogenate)). It is also noteworthy that extirpation of the terminal abdominal segments did not guarantee intact RNA preparations from worker *N. pubens*. Only with complete removal of the abdomen did acceptable RNA preparations result from workers consistently. Logically, it was assumed that contents from the venom or Dufour's gland, known from the Formicinae to contain formic acid as well as a variety of molecules (Hefetz and Blum 1978; Holldobler and Wilson 1990; Morgan et al. 2005), may be the source for the RNA degradation. The venom gland apparatus of related formicine ants (e.g., *Camponotus pennsylvanicus*) is comprised of a storage sac and long, sinuous filaments thought to collect molecular precursors for venom synthesis (Hermann, Baer, and Barlin 1975). If *N. pubens* possesses a similarly sinuous venom gland apparatus, it may explain the inconsistent results observed when the terminal abdominal segments were excluded. Specifically, not all gland components were always removed, because they often reached anteriorly into the abdomen.

**Figure 3.**
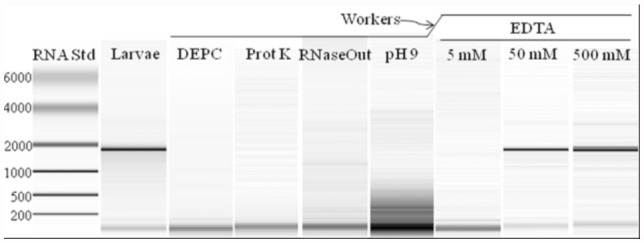
Microfluidic analyses of total RNA prepared by the Trizol method from *Nylanderia pubens* larvae (lane 2) and adults (lanes 3–9) under different conditions as indicated. High quality figures are available online.

Initially, an RNase in the adult stage was thought to be liberated during homogenization and cause RNA degradation, but guanidium isothiocyanate (Trizol in this study) is inhibitory to RNases (Sambrook and Russell 2001), and live ants were being placed directly into the Trizol and homogenized without delay. Furthermore, homogenization of workers in DEPC or proteinase K, chemicals destructive to proteins, including RNases (Pasloske 2001), and addition of RNase Out, a commercial product that inhibits RNases, did not improve the quality of RNA extracted from worker ants (Figure 3). Several attempts to identify different protein profiles between homogenates prepared from the terminal abdominal segments, whole abdomen, or abdomen with the terminal abdominal segments removed by SDS-PAGE were not informative.

Also considered was the possibility that endogenous formic acid was catalyzing the hydrolysis of RNA. However, neutralization of worker ant homogenates to pH 7 or 9 with Tris buffer also yielded degraded RNA (Figure 3). Indeed, RNA degradation was not observed to occur when synthetic formic acid (0.1 M and 1 M) was added directly to the head and thorax of adult workers or larvae before homogenization and RNA extraction. Thus, endogenous formic acid did not appear to directly cause the RNA degradation observed in adult ants.

A final series of experiments examined the effect of EDTA on RNA purification and integrity. Whole worker ants homogenized in at least 50 mM EDTA yielded intact RNA when subsequently extracted with Trizol reagent (Figure 3). Interestingly, at concentrations below 50 mM EDTA (for 10 worker ants), the resulting RNA was degraded.

Although not specifically identified, the RNA-degrading entity appears to be endogenous and liberated upon homogenization of *N. pubens* workers. This entity does not appear to be a canonical RNase, as DEPC, RNase Out (both RNase inhibitors), and proteinase K did not provide protection from its RNA-degrading activity. It also does not appear that formic acid played a role, because addition of synthetic formic acid did not result in degraded RNA from larvae or abdomenless adult ants. Transesterification of RNA can be catalyzed by nitrogenous bases (Bibillo, Figlerowicz, and Kierzek 1999), a variety of which are found frequently in venom and Dufour's glands of many ant species, including formicines (Holldobler and Wilson 1990). However, the fact that EDTA, at certain concentrations, prevented RNA degradation in workers suggests that a metal cation may be responsible, directly or indirectly, for hydrolyzing RNA. Metal cations of different valence have been reported to directly catalyze the hydrolysis of nucleic acids (particularly RNA) and indirectly by serving as structural stabilizing or integral cofactors for ribozymes, RNA molecules that catalyze the hydrolysis of RNA (Pyle 1993; AbouHaidar and Ivanov 1999; Takagi et al. 2001; Yang, Lee, and Nowotny 2006). Indeed, Mg^2+^ has been shown to serve as a Lewis acid promoting activation of the nucleophile RNA, causing its rapid degradation (AbouHaidar and Ivanov 1999; Takagi et al. 2001).

AbouHaidar and Ivanov (1999) have shown that Mg^2+^ can be a powerful catalyst for the degradation of RNA, and that EDTA inhibits its hydrolytic action. Mg^2+^ also plays an important role in ribozyme-catalyzed degradation of RNA (Takagi et al. 2001).

Although this study was unable to identify the entity or location in worker *N. pubens* causing RNA degradation, its presence is undeniable. Because *N. pubens* has emerged as an important pest ant species in recent years, and will likely be the focus of intense research effort in coming years, this study aimed to create awareness of the RNA-degrading entity in the adult stage. Fortunately, the RNA-degrading effect can be mitigated by the inclusion of EDTA in appropriate concentration. Unfortunately, EDTA carryover can inhibit subsequent RT-PCR and PCR analyses. Extraction of sound, intact RNA is a crucial first step in any downstream molecular analysis of genes. Stage-dependent degradation of RNA observed in *N. pubens* illustrates the importance of proper assessment of the integrity of nucleic acid purification processes, especially in untested organisms. Furthermore, proper techniques must be employed to make those determinations. For example, spectrophotometric examination alone may provide a deceiving assessment of the integrity of an RNA sample, as was observed in *N. pubens* workers.

## Editor's note

A recent paper by Gotzek et al. (2012) has renamed Nylanderia pubens as Nylanderia fulva.
